# Active anti-predator behaviour of red titi monkeys (*Plecturocebus cupreus*)

**DOI:** 10.5194/pb-6-59-2019

**Published:** 2019-06-05

**Authors:** Sofya Dolotovskaya, Camilo Flores Amasifuen, Caroline Elisabeth Haas, Fabian Nummert, Eckhard W. Heymann

**Affiliations:** 1 Behavioural Ecology and Sociobiology Unit, German Primate Center, Göttingen, Germany; 2 Primate Genetics Laboratory, German Primate Center, Göttingen, Germany; 3 Estación Biológica Quebrada Blanco, Quebrada Blanco, Río Tahuayo, Peru; 4 Department of Sociobiology/Anthropology, University of Göttingen, Göttingen, Germany

## Abstract

Due to their inconspicuous behaviour and colouration, it has been assumed that titi monkeys' main anti-predator behaviour is passive crypsis and hiding. So far, active predator mobbing has been documented only for black-fronted titi monkeys, *Callicebus* *nigrifrons*. Here we report for the first time mobbing behaviour of red titi monkeys, *Plecturocebus cupreus* (previously *Callicebus cupreus*), as reaction to an ocelot (*Leopardus pardalis*) and a *Boa constrictor*. We also report other active anti-predator behaviours, such as alarm calling and approaching, as reactions to tayras (*Eira barbara*) and raptors. Our observations provide additional evidence for sex differences in anti-predator behaviour, possibly related to the evolution and maintenance of social monogamy.

## 1 Introduction

Although predation is thought to play a major role in the evolution of primate behaviour and ecology, predation on primates is rarely directly observed in the wild. Encounters with potential predators, however, are observed more often, and reactions of primates to the presence of potential predators might help to estimate the extent of predation pressure by different types of predators.

Behavioural responses to predators vary considerably in primates depending on both predator and prey species. Types of responses can be broadly classified into two groups: passive (e.g. avoidance, fleeing, or hiding) and active (e.g. alarm calling, attacking, or mobbing), and they vary systematically within species depending on the type of predator (Ferrari, 2009). Active responses such as alarm calling and mobbing (the latter defined as repeated and aggressive advances on a predator accompanied by calling and displaying in a conspicuous manner; Dutour et al., 2016) often involve several or all group members and have been documented in various primate species (for example, gelada baboons – Iwamoto et al., 1996; moustached tamarins – Shahuano Tello et al., 2002; chimpanzees – Boesch and Boesch-Achermann, 2000). In many species, both pair living and living in multi-male and multi-female groups, males are more involved in these active anti-predator behaviours than females (Isbell, 1994). This special male role against predators has been suggested to account both for the evolution of multi-male primate groups and for the evolution and maintenance of pair living and pair bonding in pair living species (e.g. Crook and Gartlan, 1966; De Luna et al., 2010; van Schaik and Dunbar, 1990).

**Table 1:** Predation and predation attempts on titi monkeys.

Prey species	Predator	Type of observation	Source
*Plecturocebus*	*Eira barbara* (tayra)	Unsuccessful attacks	De Luna et al. (2010)
*discolor*	*Boa constrictor*	Successful predation	Cisneros-Heredia et al. (2008)
(*Callicebusdiscolor*)	*Harpia harpyja* (harpy eagle)	Successful predation	De Luna et al. (2010)
*Plecturocebus*	*Sapajus apella* (*Cebus apella*)	Successful predation	Sampaio and Ferrari (2005)
	(capuchin monkey)		
*Plecturocebus toppini*	*Boa constrictor*	Successful predation	Lawrence (2007)
*moloch*	*Morphnus guianensis* (crested eagle)	Successful predation	Terborgh (1983); Wright (1984)
(*Callicebus*	*Spizaetus tyrannus* and *Spizaetus ornatus*	Successful predation	Terborgh (1983); Wright (1984)
	(ornate hawk-eagles)		
*moloch*)	*Sapajus macrocephalus* (*Cebus apella*)	Successful predation	Lawrence (2007)
	*Leopardus pardalis* (ocelot)	Unsuccessful attack	Wright (1984)
	*Accipiter bicolor* (bicoloured hawk)	Unsuccessful attack	Wright (1984)
*Plecturocebus sp.*	*Harpia harpyja* (?)	Successful predation	Curtis Freese, personal communication
(*Callicebus moloch*)			with Kinzey et al. (1977)

Titi monkeys are small (body mass around 1 kg), cryptically coloured diurnal Neotropical primates living in small family groups containing an adult pair and 1–3 offspring (Bicca-Marques and Heymann, 2013). The infants are carried almost exclusively by adult males (Wright, 1984). Due to both their small body size and low group size, titis are faced with a wide range of predators (Table 1).

Because of their generally inconspicuous behaviour and colouration, it has been long assumed that the titi monkeys' main anti-predator behaviour was passive crypsis and hiding (Ferrari, 2009). More recently, however, active anti-predator group behaviours such as alarm calling and approaching have been documented in the following titi monkeys: *Plecturocebus discolor* (Cisneros-Heredia et al., 2008; De Luna et al., 2010) and *Plecturocebus moloch* (Sampaio and Ferrari, 2005). Mobbing behaviour in titis has been so far documented only for the black-fronted titi monkey (*Callicebus nigrifrons*) as reaction to tayras and an unidentified spotted cat (Cäsar et al., 2012).

During a field study on the ecology and mating system of red titi monkeys, *Plecturocebus cupreus* (previously *Callicebus cupreus*), we witnessed several encounters with different predators or potential predators and recorded the responses of the titis. Here we report, for the first time, mobbing behaviour of titis as reaction to an ocelot (*Leopardus pardalis*) and a *Boa constrictor*. We also report other active anti-predator behaviours, such as alarm calling and approaching, and discuss the more active male involvement in these behaviours.

## 2 Materials and methods

The study took place at the Estación Biológica Quebrada Blanco (EBQB), in north-eastern Peruvian Amazonia (4${}^{\circ }$21${}^{\prime }$ S, 73${}^{\circ }$09${}^{\prime }$ W). It is embedded in primary tropical rainforest of the Tierra Firme type and has included a small area with anthropogenic secondary forest regeneration since 2001. As part of a study on the mating system and ecology, we habituated and studied seven groups of titi monkeys (Group 1 has been habituated and studied intermittently since 1997) between June–December 2017 and June–December 2018. We followed the monkeys from the early morning, when the animals left a sleeping site (or from when we could locate the group), until the late afternoon, when the animals retired to a sleeping site. Each group was followed by a team of two observers. We documented encounters with predators opportunistically, recording the time, predator taxon when possible, and monkey behaviour during and after the encounters. The total observation time was approximately 2750 h (from 387 to 520 h for each of the groups mentioned in this study). During the encounters observed in 2017, Group 1 comprised an adult male and female, one subadult male, and one juvenile male; in 2018, it comprised adult male and female, one subadult male, and an infant carried by the adult male. Group 2 comprised an adult male and female during all the encounters mentioned here. Group 4 comprised an adult male and female and a juvenile during all the encounters mentioned here. Group 6 comprised an adult male and female, subadult male, and a juvenile during the encounter mentioned here.

### Statement of ethics

All research protocols reported in this paper were reviewed and approved by the German Primate Center and the Servicio Nacional Forestal y de Fauna Silvestre (SERFOR) of the Peruvian Ministry of Agriculture (MINAGRI; permit no. 249-2017-SERFOR/DGGSPFFS). All research reported in this paper adhered to the legal requirements of the country in which the work took place.

## 3 Results

In total, we observed 12 encounters with potential predators. We also observed nine encounters with squirrel monkeys (*Saimiri cassiquiarensis*); since titis showed anti-predator response during these encounters, we include them in our report (see discussion below).

### 3.1 Ocelot encounter

On 17 June 2017 at 06:50 GMT - 5, Group 1 was moving through the canopy at a height of approximately 10 m. While passing through a big tree, the adult male started alarm calling and was joined after approximately 2–3 min by all other group members. We then noticed an ocelot (*Leopardus pardalis*) lying on a big branch of the same tree. The titis started mobbing the ocelot: all the group members surrounded it and began to move erratically around it, emitting alarm calls, lashing tails, swaying heads, and showing piloerection. The titis were moving rapidly towards and away from the ocelot but never approached closer than 2–3 m. The ocelot did not make any moves towards the titis and kept lying on the branch but had noticed the human observers and was staring at us from above. The titis kept mobbing the ocelot and vocalizing until 07:38, when they left the tree. The ocelot stayed on the same branch without moving. The titis then travelled approximately 50 m, still emitting alarm calls every 3–5 min. At 07:55, however, all the group members returned to the same tree and started mobbing the ocelot again, vocalizing, tail lashing, and moving rapidly towards and away from it. At 08:03, the group finally left the tree and proceeded to a feeding tree. The ocelot was still lying on the same branch when the titis left.

### 3.2 Boa encounter

On 14 July 2017, at 10:00, Group 1 was moving through the canopy at a height of approximately 20 m. At a height of approximately 25 m, a boa (*Boa constrictor*) was lying curled up on a branch. All group members started to produce alarm calls and to mob the boa, jumping through the canopy around it, making erratic movements towards and away from the snake, tail lashing and showing piloerection but never approaching the snake closer than 2–3 m. The mobbing lasted until 10:10, when the titis left the tree and moved on through the canopy at a height of approximately 20 m.

### 3.3 Tayra encounters

On 11 October 2017 at 06:10 Group 2 entered a fruiting tree and started feeding on fruits. At 06:13, the male started alarm calling; a tayra (*Eira barbara*) was lying in a nearby tree approximately 7 m from the titis. The male continued vocalizing until 06:34, staring at the tayra and from time to time moving towards it, but never approaching it closer than 5 m. The female continued feeding after the male had started alarm calling and only occasionally joined the male in vocalizing, emitting alarm calls for several seconds. At 06:34, the tayra left the area. The titis then moved to a nearby tree and rested out of view in a vine tangle until 07:30.

On the same day, at 14:30, Group 2 started climbing up a tree approximately 50 m from the site of the previous encounter with the tayra. At the same time, we saw a tayra moving down the same tree. When the titis noticed the tayra, they turned downwards and moved away quickly while emitting alarm calls. After travelling approximately 30 m, they ran into a group of squirrel monkeys. The titis turned again and fled quickly; we could not find the group again until 15:40.

### 3.4 Birds of prey encounters

On 19 August 2017, at 10:31, Group 6 was scattered around, foraging at a height of 5–10 m. A juvenile grey-headed kite (*Leptodon cayanensis*) landed on a tree nearby. The adult male gave alarm calls for about 30 s, tail lashing and displaying piloerection. Until 10:35, when the kite left, the male stayed vigilant and gave one more short alarm call.

On four other occasions in July and August 2018, the adult male and female of Group 4 gave alarm calls simultaneously after encounters with unidentified birds of prey, vocalizing for 1 to 8 min.

On 19 September 2018, Group 1 was resting in a tree at a height of approximately 10 m; the female was separated by more than 10 m from the other group members. At 07:56, an unidentified hawk flew in and perched on another tree approximately 10 m from the titis. The adult male, who was carrying an infant, started alarm calling. At 08:06, the hawk attacked the subadult male. The adult male called again for 2 min and moved downwards into a denser part of the tree crown. At 08:18, the hawk attacked the subadult again, and all group members including the female, who had joined the group by then, emitted alarm calls. Shortly after, the hawk left the area. Then the titis rested for about 20 min.

### 3.5 Capuchin and squirrel monkey encounters

On two occasions in July 2017, Group 1 fled quickly and hid in the vine tangle after hearing large-headed capuchin (*Sapajus macrocephalus*) calls from the distance. On nine occasions in 2017 and 2018, Group 2 fled and hid from squirrel monkey (*Saimiri cassiquiarensis*) groups; on four occasions, Group 2 could not be found for up to 2 days after the encounter with squirrel monkeys. However, we never observed the titis emitting alarm calls in encounters with capuchins or squirrel monkeys.

## 4 Discussion

### 4.1 Active anti-predator behaviour of red titi monkeys

Here, we observed mobbing behaviour in *P. cupreus* as a reaction to an ocelot and *Boa constrictor*. Until now, mobbing has not been described for *P. cupreus* but was reported for another titi monkey species, *C. nigrifrons*, as a reaction to tayras and an unidentified spotted cat (Cäsar et al., 2012). We also observed other active predator responses such as alarm calling and approaching predators; these findings are in agreement with reports on the same behaviours in *C. discolor* (Cisneros-Heredia et al., 2008; De Luna et al., 2010) and *C. moloch* (Sampaio and Ferrari, 2005).

The mobbing of ocelot observed here lasted for almost 40 min; soon after leaving the area, the titis returned and continued to mob the predator again for almost 10 min. Interestingly, during both the ocelot and the *Boa constrictor* encounters, the predators did not make any attempts to attack the monkeys or even move in their direction; both the ocelot and the snake remained exactly at the same spot throughout the encounter, and the ocelot was watching human observers much more intently than the monkeys. Moreover, none of the trees where the predators were encountered had been used as feeding or sleeping trees by the titi monkey group.

One of the adaptive bases for predator mobbing has been hypothesized to be reducing the probability that a predator will attack or remain in the area (Curio, 1978). As both ocelots and snakes hunt by ambush and rely on crypsis and surprise for successful predation, mobbing could decrease the probability of a successful ambush by removing the advantage of a surprise attack, either discouraging the predators from attacking or inducing them to leave the area. This might be the reason for the titis to actively mob the predators in the trees they were merely passing by and, in the case of the ocelot, to even come back for a second round of mobbing. This might also be the reason to mob tayras; although never observed in our study, the mobbing of tayras has been reported for *C. nigrifrons* (Cäsar et al., 2012) and other Neotropical primates (Cäsar and Zuberbühler, 2012). Although tayras, unlike ocelots or snakes, never ambush their prey, they can stalk it (Presley, 2000), and thus mobbing could have the same effect on them as on ocelots or snakes. It is noteworthy that during the encounters described here, neither the ocelot nor the snake left the area as a result of mobbing; but they also did not try to attack the titis.

It is assumed that small-bodied primates with cryptic pelage colouration would mainly rely on passive anti-predator strategies such as hiding or fleeing. However, there are multiple reports on mobbing behaviour of miniature callitrichids in response to tayras (common marmoset, *Callithrix jacchus* – Bezerra et al., 2009), buffy-headed marmosets (Ferrari and Ferrari, 1990), snakes (pygmy marmosets, *Cebuella pygmaea* – Soini, 1988) and moustached tamarins (*Saguinus mystax* – Shahuano Tello et al., 2002). In slightly larger titis and owl monkeys, active anti-predator responses seem to be quite rare, with only few reports available (owl monkeys, *Aotus azarae* – Savagian and Fernandez-Duque, 2017; *C. nigrifrons* – Cäsar et al., 2012). This might be explained by the differences in group size, with the range of 4–20 in callitrichids and only 2–7 in titi and owl monkeys. The small groups' size and relatively large proportion of immature individuals in titi and owl monkeys probably mainly reinforce cryptic rather than active anti-predator responses (Ferrari, 2009).

Interestingly, although titis are known to produce referential alarm calls, with acoustically distinct variants given to terrestrial or aerial threats (Cäsar and Zuberbühler, 2012), we could not differentiate between different types of calls by listening.

### 4.2 Active male involvement in the anti-predator behaviour

During 4 out of 10 encounters described here, the adult males were involved in active anti-predator behaviours more than other group members: the alarm calling was initiated by adult males and joined by other group members only later. During the tayra encounter, the male was calling and approaching the tayra for more than 20 min, while the female continued feeding and only shortly joined the male in alarm calling several times. The females, in contrast, did not initiate the alarm calling or other anti-predator responses in any of the observed encounters. Similar findings were reported by De Luna et al. (2010), who observed adult males initiating alarm calling in encounters with tayras.

In contrast to De Luna et al. (2010), where active anti-predator behaviours were only observed in the presence of infants, only one of the encounters described here happened in the presence of an infant (unidentified hawk, 19 September 2018). On this occasion, the male carrying the infant, although emitting alarm calls, did not approach the predator but hid in the dense part of the tree. All other encounters, including two involving mobbing a predator, happened in the absence of infants.

One of the hypotheses for the evolution and maintenance of pair living and pair bonding suggests that they developed as a result of selection for male services, such as direct infant care, protection against predators, or infanticide prevention (e.g. van Schaik and Dunbar, 1990). So far, it is unclear if any significant risk of male infanticide exists in titi monkeys. However, extensive male care combined with more active male involvement in defence in titi monkeys suggests that male services such as direct infant care and protection against predators might have played a role in evolution or maintenance of pair living and pair bonding in this species.

### 4.3 Reactions to capuchin and squirrel monkeys

On multiple occasions, we observed the titis fleeing and hiding from squirrel monkey (*S. cassiquiarensis*) groups. It might appear to be an odd behaviour, since squirrel monkeys are mainly frugivorous and insectivorous and have never been reported hunting on primates (Defler, 2004). However, squirrel monkeys often form mixed species troops with capuchin monkeys (Podolsky, 1990). Capuchin monkeys are known to capture mammals (Fedigan, 1990; Fragaszy et al., 2004) and have been directly observed hunting titi monkeys (Lawrence, 2007; Sampaio and Ferrari, 2005). Fear reaction to squirrel monkeys is probably explained by titi monkeys associating the presence of squirrel monkeys with the presence of capuchin monkeys. A similar avoidance of and fleeing from squirrel monkeys has been observed in moustached (*Saguinus mystax*) and black-fronted tamarins (*Leontocebus nigrifrons*) at EBQB (Eckhard W. Heymann, personal observations, 2019).

Interestingly, we never observed the titis emitting alarm calls in encounters with capuchin or squirrel monkeys. Since capuchin monkeys, unlike cats or snakes, do not hunt by ambush, passive response such as fleeing and hiding might be more advantageous in encounters with this predator.

## 5 Conclusions

Active anti-predator responses described here indicate that, despite their generally cryptic behaviour, titi monkeys' responses to predators are not always passive, as had been long assumed. During 4 out of 10 encounters, adult males showed more active anti-predator responses than females or other group members. Our observations put the previous suggestion by De Luna et al. (2010) on a broader base, namely that protection against predators might have played a role in evolution and maintenance of pair living and pair bonding in this species.

## Data Availability

Data sharing is not applicable to this article as this study analyses qualitative data and no datasets were generated during this study.
